# Biomarkers That Currently Affect Clinical Practice in Lung Cancer: EGFR, ALK, MET, ROS-1, and KRAS

**DOI:** 10.3389/fonc.2014.00204

**Published:** 2014-08-11

**Authors:** Grzegorz J. Korpanty, Donna M. Graham, Mark D. Vincent, Natasha B. Leighl

**Affiliations:** ^1^Division of Medical Oncology and Hematology, Princess Margaret Cancer Centre, University of Toronto, Toronto, ON, Canada; ^2^London Regional Cancer Program, Department of Medical Oncology, London Health Sciences Centre, London, ON, Canada

**Keywords:** EGFR, ALK, Met, ROS-1, KRAS, lung adenocarcinoma, biomarkers

## Abstract

Lung cancer remains the most lethal malignancy in the world. Despite improvements in surgical treatment, systemic therapy, and radiotherapy, the 5-year survival rate for all patients diagnosed with lung cancer remains between 15 and 20%. Newer therapeutic strategies rely on specific molecular alterations, or biomarkers, that provide opportunities for a personalized approach to specific patient populations. Classification of lung cancer is becoming increasingly focused on these biomarkers, which renders the term “non-small cell lung” cancer less clinically useful. Non-small cell lung cancer is now recognized as a complex malignancy and its molecular and genomic diversity allows for patient-centered treatment options. Here, we review advances in targeted treatment of lung adenocarcinoma with respect to five clinically relevant biomarkers – *EGFR*, *ALK*, *MET*, *ROS-1*, and *KRAS*.

## Introduction

Lung cancer remains one of the most commonly diagnosed malignancy worldwide and the leading cause of cancer-related death (1). Until the last decade, non-small cell lung cancer (NSCLC) was considered a single disease, and systemic treatment of metastatic NSCLC was limited to platinum-based chemotherapy doublets resulting in approximately 20% response rates and median survival of 8 months (2). Only recently, we have realized that recognition of histological subtypes of NSCLC is clinically relevant when choosing systemic, platinum-based chemotherapy (3). In recent years, the oncology community has seen a paradigm shift in the molecular diagnosis and treatment of lung cancer thanks to identification of sensitizing mutations within the epidermal growth factor receptor gene *(EGFR)* to EGFR tyrosine kinase inhibitors (EGFR-TKIs) (erlotinib, gefitinib, and afatinib), and anaplastic lymphoma kinase gene *(ALK)* rearrangements (i.e., *EML4-ALK*) to ALK inhibitors (crizotinib and ceritinib) (4).

These breakthrough discoveries provide the unique opportunity for molecularly selected lung cancer patients to receive targeted, personalized treatment options that translate into clinically meaningful benefit (4). Molecular testing of NSCLC is now widely recommended by oncology societies because it provides personalized treatment options and better outcomes for patients with metastatic disease (5, 6).

To improve outcome, molecular profiling of lung cancer tumors should be available to all NSCLC patients in order to make targeted therapy available to patients with actionable/“druggable” driver mutations (7–9). Currently, we can offer these treatments routinely to patients with *EGFR*-mutated and *ALK*-rearranged NSCLC, the vast majority of whom have adenocarcinoma histology.

This review summarizes the most recent data on efficacy, risks, and benefits of novel biologic therapies in NSCLC focusing on *EGFR, ALK, MET, ROS-1*, and *KRAS* (Table 1).

**Table 1:** **Clinically relevant biomarkers in NSCLC**.

Biomarker	Treatment	Genomic aberration	Prevalence in NSCLC patients	Reference
EGFR	1. Tyrosine kinase inhibitors (e.g., gefitinib, erlotinib, and afatinib) 2. Monoclonal antibodies (e.g., cetuximab and necitumumab)	1. Activating mutation within intracellular catalytic domain of *EGFR* 2. Over-expression of extracellular part of EGFR	*EGFR m*utations (non-squamous histology) 1. ~15% in Caucasians 2. ~40% in Asians 3. ~75–80% in never-smoker Asians *EGFR* mutations (squamous histology) 1. ~5% EGFR over-expression 1. 39% in adenocarcinoma 2. 58% in squamous cell carcinoma 3. 38% in large-cell carcinoma	(10–14)
ALK	Tyrosine kinase inhibitors (e.g., crizotinib and ceritinib)	Chromosomal translocation and fusion of *ALK* gene	1. 3–5% in unselected NSCLC 2. ~10% in non-never-smokers 3. <1% in squamous carcinoma	(15–19)
MET	1. Tyrosine kinase inhibitors (e.g., tivantinib, cabozantinib, and crizotinib) 2. Monoclonal antibodies (onartuzumab, AMG 102, ficlatuzumab)	1. Increased *MET* copy number 2. Over-expression of extracellular part of MET receptor	1. 2–4% *MET* amplification (untreated) 2. 5–20% *MET* amplification in EGFR-TKI-resistant tumors 3. 25–75% over-expression of extracellular part of MET receptor	(20–23)
ROS-1	Tyrosine kinase inhibitor (crizotinib)	Chromosomal translocation and fusion of *ROS-1* gene	1–2% in unselected population	(24–27)
KRAS	Downstream pathway inhibitors (e.g., MEK inhibitors selumetinib and trametinib)	Activating mutation within catalytic *RAS* domain	1. *KRAS* are rare in never-smokers 2. ~25–30% in adenocarcinoma 3. ~5% in squamous cell carcinoma	(28–37)

## EGFR

The epidermal growth factor receptor family (ERBB family) comprises four tyrosine kinase receptors: HER-1 (EGFR), HER-2/neu (ERBB2), HER-3 (ERBB3), and HER-4 (ERBB4) (38, 39). Following ligand-binding, EGFR receptors homo- and hetero-dimerize and promote autophosphorylation of the intracellular tyrosine kinase domain and initiate molecular cascade of events involved in growth, cell proliferation, differentiation, and survival (10, 11, 40). Small-molecule receptor tyrosine kinase inhibitors (TKIs) bind to the intracellular catalytic domain of the tyrosine kinase and inhibit receptor autophosphorylation and activation of downstream signaling pathways by competing with adenosine triphosphate (ATP) (41). Gefitinib and erlotinib are the most extensively studied reversible EGFR-TKIs in patients with metastatic NSCLC (42, 43). The majority of unselected NSCLC patients will not respond to treatment with EGFR-TKIs. Patients of Asian ethnicity, females, never-smokers, or those with adenocarcinoma histology, were initially identified as a population with the most substantial clinical benefit from EGFR-TKIs (12, 44–53). The marker of sensitivity to EGFR-TKIs was unknown until 2004 when activating mutations in exon 18, 19, and 21 of the *EGFR* gene were discovered (54–56). The majority of mutations are either point mutations leading to amino acid substitutions (exon 18 and 21) or in-frame deletions (exon 19) clustered around the ATP-binding pocket of the intracellular tyrosine kinase domain (13). A kinetic analysis of the intracellular domains of mutant *EGFR* has shown that the mutant receptor compared with a wild-type shows reduced affinity for ATP in the presence of EGFR-TKI (57).

The Iressa Pan-Asia Study (IPASS) was the first phase III randomized trial that demonstrated superior outcome with first-line EGFR-TKI treatment in patients with *EGFR*-mutant NSCLC when compared with platinum-based chemotherapy in a retrospective subgroup analysis (58). Other trials have employed a similar approach to the IPASS study and reported similar results (59, 60).

Four randomized phase III trials prospectively compared the efficacy of first generation EGFR-TKIs against standard platinum-based chemotherapy in patients with *EGFR* mutation-positive NSCLC (61–67). In all four trials, *EGFR*-mutated NSCLC patients treated with TKIs (erlotinib or gefitinib) had significantly better ORR, PFS, and quality of life (QOL) when compared with patients treated with platinum-based chemotherapy (58, 61, 63, 65, 67–70). Despite significant PFS benefit of EGFR-TKIs in *EGFR*-mutant NSCLC patients, none of the trials showed statistically significant survival improvement, which is likely related to a high rate of patient crossover to EGFR-TKI from first-line chemotherapy upon progression or development of acquired resistance.

Afatinib is a second-generation EGFR-TKI that irreversibly blocks EGFR and Her-2 (71, 72). LUX-Lung 3 was a phase III clinical trial of afatinib compared to cisplatin-pemetrexed chemotherapy in treatment-naïve patients with *EGFR*-mutant advanced lung adenocarcinoma (73). Both median PFS and ORR were significantly better in patients treated with afatinib compared with chemotherapy. A pooled, retrospective subgroup analysis of LUX-Lung 3 and LUX-Lung 6 trial at 2014 ASCO annual meeting demonstrated better OS for patients with *EGFR* exon 19 deletion vs. *EGFR L858R* exon 21 insertion mutations (HR = 0.59; CI 0.45–0.77; *p* < 0.001 vs. HR = 1.25; CI 0.92–1.71; *p* = 0.16) (74). First-line treatment of *EGFR* mutation-positive NSCLC with EGFR-TKIs (gefitinib, erlotinib, and afatinib) is now recommended worldwide (5, 9). AZD9291 and CO-1686 are irreversible selective EGFR inhibitors, which demonstrate significant activity in patients with acquired resistance to first-generation EGFR-TKI, and are currently under development. One of the most common mechanisms of resistance to EGFR-TKIs is the development of T790M mutation (~50% of patients), which prevents binding of reversible EGFR-TKI to the EGFR kinase domain while preserving its catalytic activity (75). In patients with tumors harboring T790M mutation, AZD9291 and CO-1686 show promising 64 and 58% ORR, respectively (76, 77).

## ALK

The *EML4-ALK* fusion gene is a product of inversion within the short arm of chromosome 2, where *ALK (anaplastic large-cell lymphoma kinase)* joins *EML4* (*echinoderm microtubule-associated protein-like 4*) to form a fusion gene (15). The product of *EML4-ALK* fusion is a chimeric protein with constitutive ALK activity and is detected in 3–6% of unselected NSCLC and especially among never-smokers or light ex-smokers who have adenocarcinoma histology (16–19). *ALK* rearrangements are nearly almost mutually exclusive with *EGFR* or *KRAS* mutations, although some rare exceptions exist (78). *ALK*-positive NSCLC represents a distinct molecular subtype that can be targeted with ALK-specific treatments (15, 24). Crizotinib is an oral small-molecule TKI that targets ALK, MET, and ROS1 tyrosine kinases (79–82). Crizotinib received accelerated US Food and Drug Administration (FDA) approval for treatment of *ALK*-positive NSCLC based on an objective response rate of 60% and median PFS of 8–10 months in single-arm studies (16, 79, 83, 84).

A first-line phase III study (PROFILE 1014) assessed efficacy of crizotinib vs. cisplatin/carboplatin-pemetrexed chemotherapy in patients with *ALK*-positive NSCLC. Recently presented data at the 2014 ASCO Annual Meeting demonstrated significantly better median PFS and ORR when compared with patients who received chemotherapy – 10.9 vs. 7.0 months and 74 vs. 45%, respectively (85). No survival benefit was demonstrated at the time of data cut-off and may never be, since patients who progressed on chemotherapy were allowed to crossover to crizotinib. The PROFILE 1007 phase III study investigated the efficacy of crizotinib vs. standard of care second-line chemotherapy (pemetrexed or docetaxel) in previously treated *ALK*-positive NSCLC (86). Patients treated with crizotinib demonstrated significantly improved median PFS when compared with chemotherapy – 7.0 vs. 3.0 months. No overall survival benefit was noted likely due to a high rate of patient crossover to the crizotinib arm from chemotherapy. Patients treated with single-agent pemetrexed had higher ORR when compared with docetaxel (29 vs. 13%).

After clinical recognition of acquired resistance to crizotinib, multiple second-generation ALK inhibitors (LKD378, AP26113, and TSR-011) entered early phase clinical trials for patients with ALK-positive solid tumors, including NSCLC (87, 88). Recently published results of a phase I clinical trial of ceritinib (LDK378) in patients with *ALK*-rearranged NSCLC demonstrated a ORR of 58% in all patients and 56% in crizotinib-resistant patients (88). Median PFS in crizotinib-naïve patients was 10.4 and 6.9 months in the crizotinib-pretreated population. Ceritinib received accelerated FDA approval in April 2014 and confirmatory trials with ceritinib in this group of patients are ongoing (http://www.fda.gov/newsevents/newsroom/pressannouncements/ucm395299).

## MET

*MET* is a proto-oncogene that encodes for the heterodimeric transmembrane MET tyrosine receptor kinase. Its only known ligand – hepatocyte growth factor (HGF) (89). Binding of HGF to the MET receptor activates the tyrosine kinase and downstream signaling pathways including PI3K/AKT, Ras-Rac/Rho, mitogen-activated protein kinase (MAPK), and phospholipase C (PLC) involved in cell motility and invasion (20, 21, 89). The MET receptor is expressed in approximately 40–50% of NSCLC tumors; high levels of receptor expression, as well as high *MET* gene copy number are independent prognostic factors of poor outcome in patients with resected NSCLC (22, 23). *MET* amplification is recognized as one of the potential molecular mechanisms of acquired resistance in *EGFR*-mutated NSCLC to EGFR-TKIs (90, 91).

Pre-clinical studies showed promising results of combined blockade of EGFR and MET signaling pathways in NSCLC (92). MET inhibitors can be divided into mAbs targeting HGF or the MET receptor (AMG 102, ficlatuzumab, and onartuzumab) or MET TKIs (tivantinib, cabozantinib, foretinib, and crizotinib) (93).

A phase II randomized study compared onartuzumab plus erlotinib vs. erlotinib alone in second- and third-line treatment. Onartuzumab, in combination with erlotinib, significantly improved PFS and OS in patients with increased *MET* gene copy (≥5) assessed by FISH (*MET*-FISH positive) as well as in patients with over-expression of MET receptor as assessed by immunohistochemistry (MET-IHC positive) regardless of gene amplification status (94). Unfortunately, a confirmatory phase III MET-Lung trial that randomized MET-IHC-positive NSCLC patients to combination onartuzumab/erlotinib vs. erlotinib alone was stopped prematurely due to lack of clinically meaningful efficacy in the combination arm (95).

Tivantinib was investigated in combination with erlotinib (EGFR-TKI) in patients with previously treated NSCLC in both phase II and phase III trials (96, 97). In the phase II trial, an exploratory subgroup analysis showed that MET-IHC-positive patients with non-squamous histology harboring *KRAS* mutations had better PFS and OS with tivantinib and erlotinib treatment when compared with erlotinib and placebo. MARQUEE, a phase III, double-blind trial randomized 1048 patients with metastatic pre-treated non-squamous NSCLC to tivantinib plus erlotinib vs. tivantinib plus placebo (98). While median PFS and ORR significantly favored tivantinib plus erlotinib (3.6 vs. 1.9 months; 10.3 vs. 6.5%, respectively), MARQUEE did not reach its primary endpoint of improved overall survival (http://eccamsterdam2013.ecco-org.eu/Scientific-Programme/Abstract-search.aspx?abstractid=6904). A subgroup analysis of patients with 2+-positive MET immunostaining demonstrated better OS, PFS, and ORR when compared to patients who had lower levels of tumoral MET expression. A further retrospective molecular subset analysis is underway to identify other potential biomarkers (*MET* copy number, *KRAS*, and *EGFR* mutations) that may help to select a target population for MET-directed treatments.

Crizotinib, which inhibits both ALK and MET, demonstrated promising results in a small pilot study (*N* = 13) of patients with *MET*-amplified NSCLC (99).

## ROS-1

ROS-1 is an orphan receptor tyrosine kinase that is phylogenetically related to ALK (100–103). *ROS-1* chromosomal rearrangements with *CD74*, *EZR, SLC24A2*, and *FIG* genes define a new genomic driver in 1–2.5% of NSCLC patients (25, 26). Clinical characteristics of NSCLC patients with *ROS-1* rearrangements are similar to patients with *ALK*-rearranged NSCLC – more commonly seen in patients of Asian ethnicity, young age (median age 49.8 years), female sex, never-smokers, and adenocarcinoma histology (25). *ROS-1* rearrangements appear mutually exclusive of other known oncogenic drivers like *EGFR*, *KRAS*, *HER-2*, *ALK*, *RET*, and *MET* aberrations (27, 104). Pre-clinical data showed activity of ALK inhibitors (i.e., crizotinib and TAE684) in *ROS-1*-rearranged NSCLC cell lines given the high degree of homology between *ALK* and *ROS-1* tyrosine kinase domains (25). This led investigators to assess the benefit of crizotinib in this unique patient subset. Efficacy has been demonstrated with an overall response rate of 56% and 6-month PFS of 71% in 25 evaluable patients (105). There are a number of currently ongoing phase I and II studies investigating activity of crizotinib, dual ALK/ROS1 inhibitor PF-06463922, and ceritinib in *ROS-1*-rearranged NSCLC.

Since *ROS-1*-rearranged NSCLC is rare and detection of *ROS1* fusions by a break-apart FISH assay is expensive and labor intensive, diagnostic algorithms and simpler screening methods (e.g., by immunohistochemistry) are needed to identify patients with *ROS-1*-rearranged NSCLC (104, 106). At this moment, patients without driver mutations like *EGFR*, *KRAS*, *HER-2*, *ALK*, and *RET* rearrangements and *MET* amplifications should be screened for *ROS-1* fusions (preferentially never-smokers) since they can be offered targeted treatment with crizotinib.

## KRAS

The *RAS* oncogene family, *HRAS*, *KRAS*, and *NRAS*, encodes intracellular transducer proteins (small GTPases) that are involved in transmitting signals from extracellular growth factor receptors like EGFR to the cell (107, 108). As G proteins, they are located on the intracellular side of the plasma membrane, bind guanine nucleotides, and have GTP-ase activity (109). In the resting state, RAS proteins are bound to GDP and are inactive. Upon exchange of GDP to GTP, the RAS-GTP complex activates multiple downstream pathways (MAPK, STAT, and PI3K) that regulate cell proliferation, motility, and apoptosis (110). After a short period, the signaling configuration of RAS is halted by intrinsic GTP-ase activity. Activating *RAS* mutations prevent GTP hydrolysis to GDP, thus the RAS protein is rendered constitutively active with uncontrolled activation of downstream signaling pathways (111).

*KRAS* mutations are present in approximately 30% of lung adenocarcinomas and less commonly in squamous NSCLC (~5%) (28). They are found more frequently in Caucasians with lung cancer than in the Asian population and in current- or ex-smokers when compared with never-smokers (29, 110). Most *KRAS* mutations in NSCLC are single amino acid substitutions in codon 12 (80%) and to a lesser extent in codons 13 and 61 (30). In current- or ex-smokers, *KRAS* mutations are usually transversions (pyrimidine nucleotide is exchanged for purine or *vice versa*; e.g., G → T or G → C) and transitions in never-smokers (purine nucleotide is exchanged for another purine or pyrimidine for another pyrimidine; e.g., G → A or C → T) (29). *KRAS* mutations are nearly always mutually exclusive with *EGFR* and *BRAF* mutations although rare co-existence of *EGFR* and *KRAS* mutations has been observed (12, 31–33). *KRAS* mutations co-exist with *PIK3CA* mutations in approximately 19% of *PIK3CA*-mutant NSCLC (32).

It has been postulated for over 20 years that *KRAS*-mutant NSCLC may be associated with poor outcome. However, multiple studies have shown conflicting results due to heterogeneity among the studies, including tumor type, stage, treatment, and study end points (28, 34). A meta-analysis of 28 studies published in 2005 demonstrated that *KRAS* mutation was a significant prognostic marker when polymerase chain reaction sequencing was used as a detection method (35). Recently published results of a LACE-Bio pooled retrospective analysis reported no prognostic or predictive (in regard to benefit from adjuvant chemotherapy) effect of *KRAS* mutations in patients with resected NSCLC (36). A subset analysis of patients with NSCLC with *KRAS* codon 13 mutations suggests that adjuvant chemotherapy may have a deleterious effect in this subgroup, but needs to be further validated (HR – 5.78; 95% CI, 2.06–16.2) (36). In the absence of prospective, large, randomized clinical trials, *KRAS* mutation status in NSCLC cannot be used as a prognostic nor predictive biomarker for treatment with exception of negative predictive value of *KRAS* mutations and response to EGFR-TKI (37, 112).

Direct inhibition of KRAS has been unsuccessful so far due to its molecular and functional complexity (113). The activation of the RAS-RAF-MEK-ERK signaling pathway as a consequence of *KRAS* mutations renders it an attractive target for small-molecule inhibition in *KRAS*-mutated NSCLC. Given the critical location in this signaling pathway, MEK has been recognized as an important target, downstream from KRAS, for anti-cancer therapy (114).

The efficacy of treatment with a combination of the orally available potent MEK inhibitor selumetinib plus docetaxel chemotherapy has been demonstrated in the treatment of patients with advanced *KRAS*-mutant NSCLC (115). Median PFS was 5.3 months in the selumetinib group and 2.1 months in the placebo group (*p* = 0.014), with a 37% ORR in the selumetinib/docetaxel arm and no response in the docetaxel alone arm (*p* < 0.0001).

Trametinib is another orally available MEK inhibitor that has been combined with docetaxel or pemetrexed in phase I/Ib trial in patients stratified by *KRAS* mutation status (116). While no difference in response rate was seen between the pemetrexed-treated groups, these response rates compare favorably with historical data for second-line chemotherapy treatment and support the absence of any negative interaction between these agents (117). Given these promising findings, ongoing studies are investigating the optimal combination of MEK inhibition and chemotherapeutic agents (www.clinicaltrials.gov).

Early studies have also suggested that the subgroup of *KRAS*-mutant NSCLC patients may benefit from targeting the PI3K-AKT-mTOR signaling pathway, downstream from KRAS. The mTOR inhibitor, ridaforolimus, has been investigated in patients with disease progression following chemotherapy with randomization to continued therapy or placebo after 8 weeks of treatment. Improved PFS was seen in the ongoing therapy group (4 vs. 2 months) with a trend toward survival benefit (18 vs. 5 months; *p* = 0.09) (118).

*KRAS* mutations in NSCLC, despite being the most common, remain the most intriguing and elusive of therapeutic targets. At present, targeted treatment is not available for KRAS-mutated NSCLC outside clinical trials. However, novel agents targeting downstream effector signaling pathways are under clinical development (119).

## Conclusion

In the addition to the emergence of histological subtypes as key factors in the treatment decision-making process for patients with advanced NSCLC, identification of certain genomic abnormalities and protein expression signatures that drive progression and metastasis of lung cancer have led to a completely new approach to treatment of NSCLC patients (120). For the first time, we recognize NSCLC as a heterogenous entity and are able to use the differences within tumors to tailor treatment with clear improvements in outcome for patients.

Biomarker-driven treatment has proven to be a major breakthrough in the modern management of lung cancer. New therapeutic modalities target specific genomic aberrations resulting in deregulation of select signaling pathways that are crucial for proliferation and metastasis of lung cancer.

There are a number of clinically and therapeutically relevant molecular changes within the lung cancer genome that can be now effectively targeted with systemic therapy in specific subgroups of patients (14). Ongoing research involving genomic efforts to elucidate further molecular subsets of NSCLC with ongoing development of biomarker-guided targeted therapies hopefully will continue to expand the therapeutic options for NSCLC patients.

**Figure 1:**
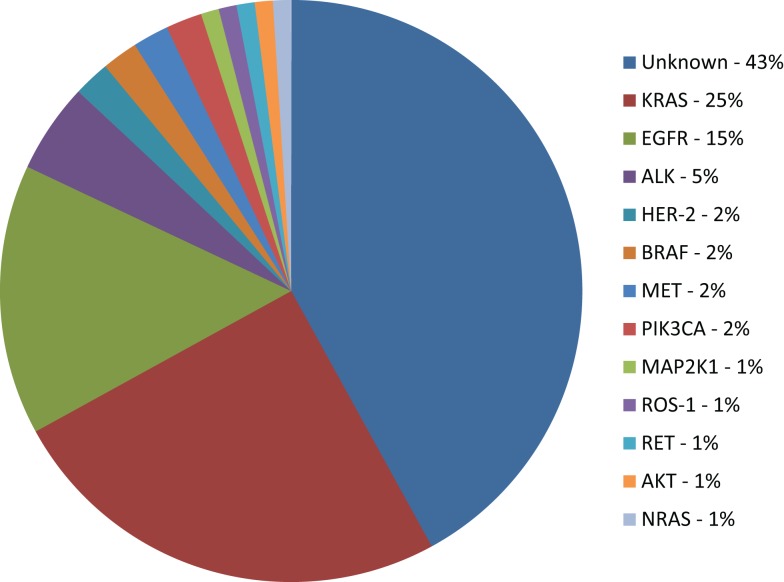
**Molecular subsets of lung adenocarcinoma**. KRAS: v-Ki-ras2 Kirsten rat sarcoma viral oncogene homolog; EGFR: epidermal growth factor receptor; ALK: anaplastic lymphoma kinase fusion; HER-2: human epidermal growth factor receptor 2; BRAF: v-raf murine sarcoma viral oncogene homolog B1; PIK3CA: phosphoinositide-3-kinase, catalytic, α polypeptide; MAP2K1: mitogen-activated protein kinase kinase 1; RET: rearranged during transfection; AKT1: v-akt murine thymoma viral oncogene homolog 1; NRAS: neuroblastoma RAS viral (v-ras) oncogene homolog (4, 9, 14, 18, 24, 31, 104).

Unfortunately, the number of patients for whom targeted therapy is suitable is still very small (Figure 1). The access to tumor tissue for biomarker assessment and *de novo* molecular and genomic tumor heterogeneity (that may be further increased during the biomarker-driven therapy) remain a serious challenge. Ongoing research in detection of cell-free circulating tumor DNA (cfDNA) and circulating tumor cells (CTCs) may become clinically relevant alternatives for tumor biopsy that will provide measurements of the total tumor burden as well as identify mutations arising during therapy that may be responsible for development of acquired resistance (121). Genomic screening of NSCLC tumors will continue to facilitate identification of molecular mechanisms of acquired resistance to targeted therapies. Ongoing translational and clinical research will facilitate a greater understanding of genomic alterations within lung cancer, with the aim of increasing benefit to wider population of lung cancer patients.

## Conflict of Interest Statement

The authors declare that the research was conducted in the absence of any commercial or financial relationships that could be construed as a potential conflict of interest.
